# Ionizing Radiation-Induced Structural Modification of Isoegomaketone and Its Anti-Inflammatory Activity

**DOI:** 10.3390/molecules30173466

**Published:** 2025-08-23

**Authors:** Euna Choi, Chang Hyun Jin, Trung Huy Ngo, Jisu Park, Joo-Won Nam, Ah-Reum Han

**Affiliations:** 1 Advanced Radiation Technology Institute, Korea Atomic Energy Research Institute, Jeongeup-si 56212, Jeollabuk-do, Republic of Korea; 2 College of Pharmacy, Yeungnam University, Gyeongsan-si 38541, Gyeongsangbuk-do, Republic of Korea

**Keywords:** isoegomaketone, (±)-8-methoxy-perilla ketone, electron beam-irradiation, anti-inflammation, nitric oxide, inducible nitric oxygen synthase

## Abstract

Isoegomaketone [(*E*)-1-(furan-3-yl)-4-methylpent-2-en-1-one; **1**] is abundant in the essential oil of *Perilla* species and exhibits various biological activities, such as anticancer and anti-inflammatory effects. In order to discover compounds with reduced toxicity or enhanced biological activity through structural modification of natural product-derived components, isoegomaketone was irradiated with an electron beam at five different doses, and (±)-8-methoxy-perilla ketone (**2**) was obtained with the highest yield of 3.8% (*w*/*w*) at 80 kGy. Its structure was identified by one-dimensional and two-dimensional nuclear magnetic resonance spectroscopy and high-resolution chemical ionization mass spectrometry. Compound **2** inhibited nitric oxide production and inducible nitric oxide synthase mRNA expression in a dose-dependent manner in lipopolysaccharide-stimulated RAW 264.7 cells. It also dose-dependently suppressed the mRNA expression of pro-inflammatory mediators such as IL-1β, IFN-β, and MCP-1, while having no significant effect on IL-6 mRNA levels. Furthermore, ELISA analysis demonstrated that **2** reduced MCP-1 protein expression but did not affect the protein level of TNF-α or IL-6. This study provides a reference for the structural analysis of compounds related to **2** by presenting NMR data acquired with chloroform-*d*, and is the first to report the anti-inflammatory properties of **2**.

## 1. Introduction

Isoegomaketone [(2*E*)-1-(3-furanyl)-4-methyl-2-penten-1-one; **1**] is a major component of the essential oil of *Perilla frutescens* [1], along with perllia ketone [1-(3-furanyl)-4-methyl-2-pentan-1-one]. Compound **1** has demonstrated various pharmacological activities, such as anticancer [2,3,4,5], wound healing [6], anti-tuberculosis [7], and anti-pulmonary inflammatory activity [8]. In our intensive study on anti-inflammatory activity of compound **1**, we demonstrated that compound **1** exerted anti-inflammatory effects by simultaneously inducing heme oxygenase-1 (HO-1) via the reactive oxygen species/p38 mitogen-activated protein kinase (MAPK)/nuclear factor erythroid 2-related factor 2 (Nrf2) pathway and suppressing the interferon-β (IFN-β)/signal transducer and activator of transcription 1 (STAT-1) pathway in RAW 264.7 cells, ultimately reducing nitric oxide (NO) and inducible nitric oxide synthase (iNOS) levels [9,10]. In addition, compound **1** delayed the onset of arthritis and alleviated arthritis symptoms in collagen antibody-induced arthritis mice [11]. On the other hand, perllia ketone has been known as a potent lung toxin in animal experiments involving sheep, cattle, and dogs [12,13,14]. However, recent studies have reported that its toxicity may contribute to pharmacological effects, such as anticancer, antifungal, and apoptosis induction in adoptive T cell therapy [15,16,17].

Structural modification using radiation technology has been applied to enhance the physicochemical properties and the biological activities of natural product-derived compounds. Compared to chemical synthesis methods, radiation technology minimizes the need for hazardous chemical reagents, enabling a more environmentally friendly process; induces rapid modifications in a short period of time, increasing production efficiency; and enables precise control of energy and dosage to induce specific modifications at specific sites in molecules, supporting large-scale production with reproducibility [18]. Irradiation produces various reactive molecular species and free radicals, including aqueous electron (e_aq_^−^), •OH, •H, •HO_2_, H_3_O^+^, OH^−^, H_2_O_2_, and H_2_ from water radiolysis [19] and solvated electron (e_S_^−^), H_2_, •H, CH_3_O•, •CH_2_OH, (CH_2_OH)_2_, H_2_CO, H^+^, and CH_3_OH_2_^+^ from methanol radiolysis [20]. The reactions of these species with compounds vary depending on the irradiation conditions, such as dose, exposure time, and the type of organic solvent. Recently, reviews on the transformation of physicochemical and biological properties of diverse natural products using radiation technology have been reported, highlighting its potential as a novel tool for developing food and pharmaceutical materials [18]. In the recent reports, gamma-irradiation of silibinin A produced two new flavonolignans, silibinosins A and B, and compared with silibinin A, silibinosin A more effectively inhibited lipopolysaccharide (LPS)-induced overproduction of inflammatory mediators and cytokines in RAW 264.7 cells [21]. Rotenoisin A, which was derived from gamma-irradiation of rotenone methanol solution, showed reduced toxicity compared to rotenone in a Parkinson’s disease cell model, suggesting potential for developing therapeutics while reducing adverse effects on normal cells [22]. As an extension of our research on the synthesis of structurally modified natural product derivatives using irradiation and the enhancement of their biological activity, we investigated the effects of irradiation on the structural transformation of isoegomaketone and evaluated the anti-inflammatory activity of the resulting radiolytic product.

## 2. Results and Discussion

### 2.1. Structural Identification of Radiolysis Product Derived from Isoegomaketone (***1***)

**Figure 1:**
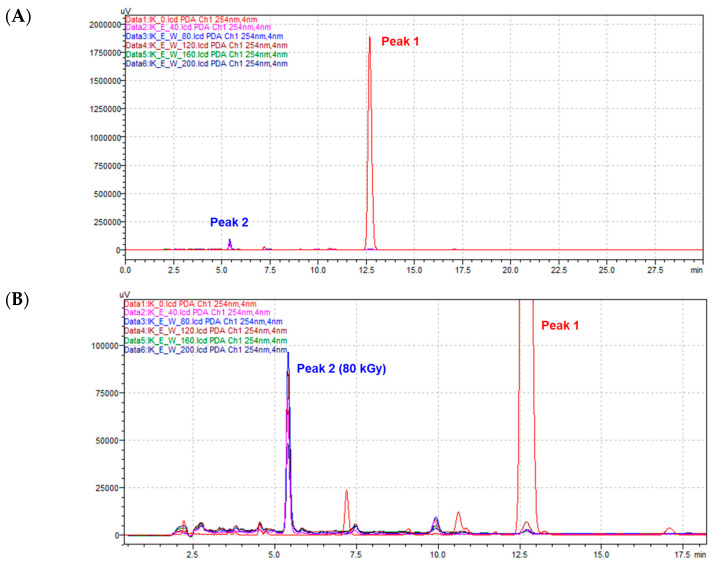
HPLC profiles (254 nm) of isoegomaketone (**1**) dissolved in water containing 10% DMSO irradiated with the following doses: 40, 80, 120, 160, and 200 kGy. (**A**) HPLC chromatogram aligned to Peak 1 (isoegomaketone, **1**). (**B**) HPLC chromatogram aligned to Peak 2 (radiolysis products of **1**).

Sample solutions containing compound **1** (99.0% purity at 254 nm) were irradiated with an electron beam at five different doses: 40, 80, 120, 160, and 200 kGy. Their radiolysis patterns were analyzed using high-performance liquid chromatography with diode-array detection (HPLC-DAD). Upon irradiation, the peak corresponding to compound **1** (*t*_R_12.7 min) disappeared, and a new peak representing the radiolysis product appeared at *t*_R_ 5.9 min (Figure 1). The peak areas and intensities of the products irradiated at five different doses showed no significant differences. However, among these doses, 80 kGy yielded the highest peak areas and intensities, and was therefore chosen as the optimal dose. This suggests that while other doses effectively promote derivative formation, 80 kGy provides the most efficient yield (Figure S1). In addition, we considered using different solvents to improve yield and tried methanol containing 10% DMSO; however, the results indicated a lower yield compared to when water containing 10% DMSO was used (Figure S2). Bulk irradiation was performed on 110 mg of compound **1**, yielding 4.19 mg of purified radiolysis product (**2**) via preparative HPLC. The practically isolated yield was calculated to be 3.8% (*w*/*w*), and the purity was verified to be 98.2% at 254 nm (Figure S3).

**Figure 2:**
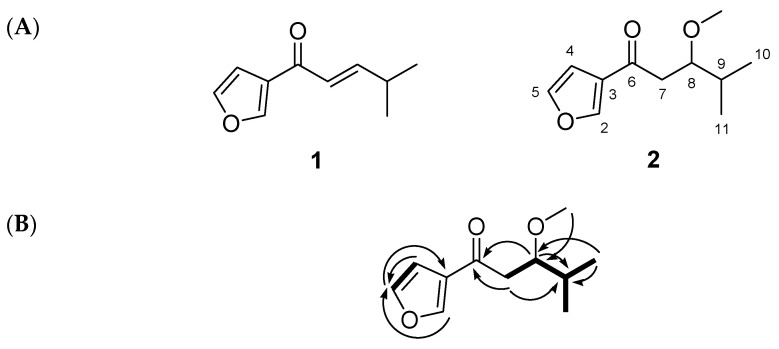
(**A**) The chemical structure of compounds **1** and **2**. (**B**) Key ^1^H-^1^H COSY (Correlation Spectroscopy, ▬) and ^1^H-^13^C HMBC (Heteronuclear Multiple Bond Correlation, →) correlations of compound **2**.

The structure of compound **2** was identified using nuclear magnetic resonance (NMR) techniques, including ^1^H, ^13^C, DEPT-135, ^1^H-^1^H COSY, ^1^H-^13^C HSQC, ^1^H-^13^C HMBC, as well as high-resolution chemical ionization mass spectrometry (HRCIMS). Compound **2** was obtained as a yellowish solid and exhibited a molecular ion peak at *m*/*z* 195.1023 [M–H]^−^ in HRCIMS, indicating a molecular formula of C_11_H_16_O_3_ (Figure S4). The ^1^H and ^13^C NMR spectra of compound **2** were similar to those of isoegomaketone [11], except for the absence of olefinic signals and the presence of new signals corresponding to an oxygenated methine at δ_H_ 3.84 (1H, dt, *J* = 7.0, 3.3 Hz, H-8)/δ_C_ 62.4 (C-8) and a methylene group at δ_H_ 3.48 (1H, d, *J* = 18.6, 7.1 Hz, H-7a) and 3.03 (1H, d, *J* = 18.6, 3.4 Hz, H-7b)/δ_C_ 34.7 (C-7). The spectra also revealed an aromatic system corresponding to a 3-furylketone moiety with signals at δ_H_ 8.18 (1H, dd, *J* = 1.2, 0.8 Hz, H-2)/δ_C_ 144.7 (C-2), 7.51 (1H, dd, *J* = 1.8, 0.8 Hz, H-5)/147.8 (C-5), and 6.83 (1H, dd, *J* = 1.8, 1.2 Hz, H-4)/108.6 (C-4), along with a carbonyl carbon at δ_C_ 191.1 (C-6). These assignments were supported by the ^1^H-^13^C HMBC correlations: H-2/C-3, C-4, C-5, H-4/C-2, C-3, C-5, and H-5/C-2, C-3, C-4 (Figure 2). Signals for an isopropyl group were observed at δ_H_ 1.10 (3H, d, *J* = 7.1 Hz, H-10)/δ_C_ 17.8 (C-10), 1.06 (3H, d, *J* = 7.1 Hz, H-11)/21.4 (C-11), and 2.72 (1H, qqd, *J* = 7.1, 3.4 Hz, H-9)/26.7 (C-9). This group was connected to the oxygenated methine and methylene groups, as evidenced by the ^1^H-^1^H COSY correlations of H-9/H-8, H-10, H-11, H-8/H-7, and H-7 and ^1^H-^13^C HMBC correlations of H-7/C-6, C-9, H-8/C-6, C-9, H-10/C-8, C-9, indicating the presence of 4-metylpentane moiety (Figure 2). Additionally, a methoxy group was assigned to C-8 based on its proton signal at δ_H_ 2.85 (3H, s, OCH_3_), which showed an ^1^H-^13^C HMBC correlation with the carbon signal at δ_C_ 62.4 (C-8). Further detailed analysis of ^1^H-^1^H COSY, ^1^H-^13^C HSQC, and ^1^H-^13^C HMBC NMR spectra enabled unambiguous assignments of all ^1^H and ^13^C NMR signals of compound **2** (Figures S5–S9). Optical rotation and electronic circular dichroism (ECD) measurements were conducted to determine the absolute configuration at C-8. However, only a slightly negative specific rotation and a nearly flat ECD curve were observed (Figure S10), suggesting that compound **2** exists as a racemate or a scalemic mixture. Accordingly, its structure was identified as (±)-1-(3-furyl)-3-methoxy-4-methylpentan-1-one, and the compound was named (±)-8-methoxy-perilla ketone.

Ina et al. discovered 1-(3-furyl)-3-methoxy-4-methylpentan-1-one with the same structure as compound **2** from the leaves of *P. fructescens* var. *acuta* in 1971 [23]. Its ^1^H NMR data were acquired in carbon tetrachloride (CCl_4_) (Table 1). In addition, 1-(3-furyl)-3-methoxy-4-methylpentan-1-one has also been synthesized by the Michael addiction reaction of isoegomaketone and methanol, by stirring isoegomaketone in methanol containing suspended Na_2_CO_3_ [24]. In that study, the ^1^H NMR data of the compound has been also acquired in CCl_4_. CCl_4_ has been considered a suitable solvent for NMR spectroscopy because it has no significant dipole moment that interferes with the NMR signals of the analytes, thereby providing a clear spectrum without solvent peaks overlapping those of the compound. However, due to its toxicity and environment impact, it is no longer used in modern chemical applications. Deuterated chloroform (CDCl_3_) is now preferred as an alternative NMR solvent. Therefore, in this study, NMR data for compound **2** were acquired using CDCl_3_, which can serve as a reference for the structural analysis of analogs related to compound **2**.

### 2.2. Anti-Inflammatory Activity of (±)-8-Methoxy-Perilla Ketone (***2***)

**Figure 3:**
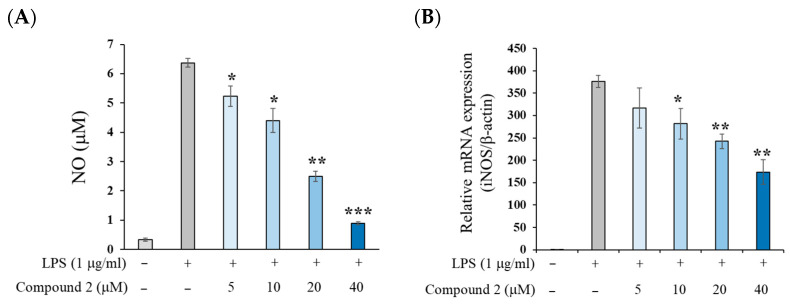
Effects of compound **2** on NO production (**A**) and iNOS mRNA expression levels (**B**) in LPS-stimulated RAW 264.7 cells. RAW 264.7 cells were treated with the indicated concentrations of compound **2** for 2 h prior to LPS addition (1 μg/mL) and were incubated for an additional 18 h. Data are presented as means ± SD (*n* = 3). * *p* < 0.05, ** *p* < 0.01, and *** *p* < 0.001 vs. the LPS-alone group.

As shown in Figure 3, treatment with LPS significantly increased NO production in mouse macrophage RAW 264.7 cells after 18 h. However, pretreatment with compound **2** resulted in a dose-dependent inhibition of NO production, with an IC_50_ value of 19.3 μM, which is approximately two-fold higher than that of the parent compound isoegomaketone (IC_50_, 8.8 μM) before electron beam irradiation [25] (Figure 3A and Figure S11). Calculation of the therapeutic index (CC_50_/IC_50_) revealed that compound **2** exhibited approximately a three-fold higher value compared to compound **1** (Figure S11). To investigate whether the NO suppression by compound **2** was associated with decreased iNOS expression, RT-PCR analysis was performed. The results revealed that iNOS mRNA expression was reduced in a dose-dependent manner following treatment with compound **2** (Figure 3B). These findings suggest that compound **2** inhibits NO production in LPS-stimulated RAW 264.7 cells through the downregulation of iNOS expression.

**Figure 4:**
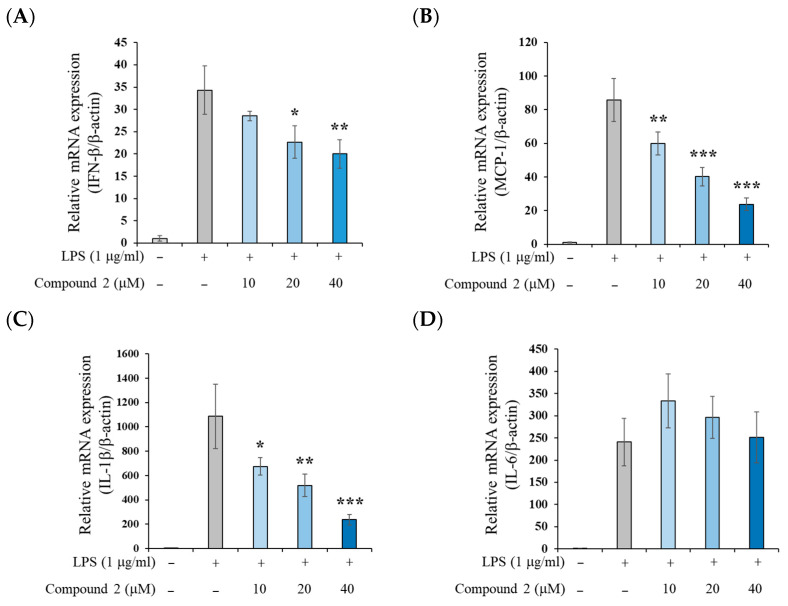
Effects of compound **2** on inflammatory cytokine mRNA expression levels in LPS-stimulated RAW 264.7 cells. (**A**) IFN-β, (**B**) MCP-1, (**C**) IL-1β, and (**D**) IL-6. RAW 264.7 cells were treated with the indicated concentrations of compound **2** for 2 h prior to LPS addition (1 μg/mL) and were incubated for an additional 4 h. Data are presented as means ± SD (*n* = 3). * *p* < 0.05, ** *p* < 0.01, and *** *p* < 0.001 vs. the LPS-alone group.

To determine whether compound **2** affects the expression of inflammatory cytokines beyond NO production in LPS-stimulated RAW 264.7 cells, we examined the mRNA levels of several pro-inflammatory mediators. As shown in Figure 4, compound **2** dose-dependently suppressed the LPS-induced mRNA expression of IFN-β, monocyte chemoattractant protein-1 (MCP-1), and interleukin (IL)-1β. However, it did not significantly alter IL-6 mRNA expression. These results suggest that compound **2** may not interfere with the myeloid differentiation primary response gene 88 (MyD88)-dependent nuclear factor kappa B (NF-κB) pathway, which is typically responsible for IL-6 induction upon LPS stimulation [26]. This finding contrasts with the parent compound **1**, which has been reported to inhibit both MyD88-dependent and independent pathways in LPS-stimulated RAW 264.7 cells [9].

**Figure 5:**
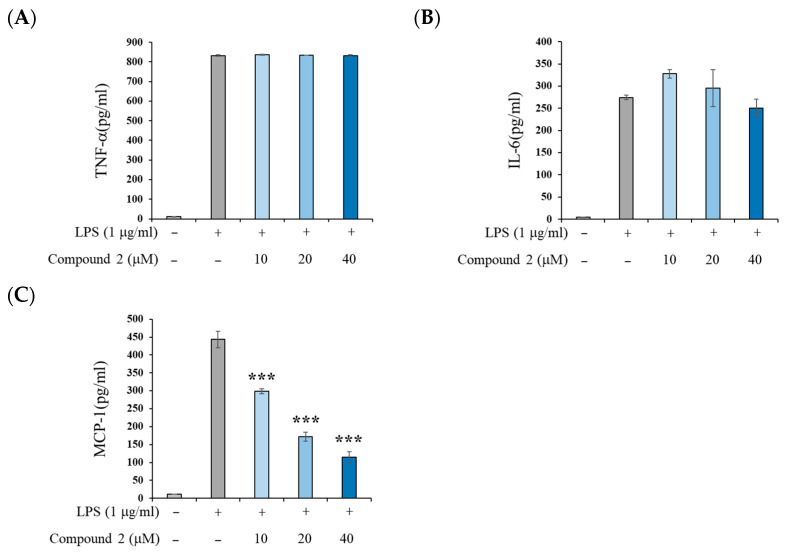
Effects of compound **2** on inflammatory cytokine protein production levels in LPS-stimulated RAW 264.7 cells. (**A**) TNF-α, (**B**) IL-6, and (**C**) MCP-1. RAW 264.7 cells were treated with the indicated concentrations of compound **2** for 2 h prior to LPS addition (1 μg/mL) and were incubated for an additional 4 h. Data are presented as means ± SD (*n* = 3). *** *p* < 0.001 vs. the LPS-alone group.

To further confirm the anti-inflammatory mechanism of compound **2**, we investigated the expression of pro-inflammatory cytokines at the protein level. As shown in Figure 5, compound **2** did not suppress the LPS-induced increase in tumor necrosis factor-α (TNF-α), and IL-6 protein levels. However, it significantly reduced MCP-1 protein expression in a dose-dependent manner. These findings are consistent with the mRNA expression data presented in Figure 4 and suggest that compound **2** does not affect the MyD88-dependent NF-κB pathway. Instead, it appears to inhibit the Toll/interleukin-1 receptor domain-containing adaptor protein inducing the IFN-β (TRIF)-dependent transcription factor interferon regulatory factor-3 (IRF3) signaling pathway, thereby reducing the expression of IFN-β and MCP-1. Although IL-1β mRNA expression was suppressed by compound **2** (as shown in Figure 4C), this effect was not confirmed at the protein level. This discrepancy is likely attributable to the fact that, in RAW 264.7 cells, LPS stimulation alone induces IL-1β mRNA expression, whereas additional stimulation with adenosine triphosphate (ATP) is required to activate the nucleotide-binding oligomerization domain (NOD)-like receptor family, pyrin domain-containing 3 (NLRP3) inflammasome, which is essential for IL-1β protein production [27]. Innate immunity provides the first line of defense against invading microbial pathogens by rapidly responding to pathogen-associated molecular patterns (PAMPs) [28]. Among its signaling cascades, the TRIF-IRF3 pathway plays a pivotal role in mediating these protective responses [29]. In contrast to compound **1**, compound **2** inhibits the TRIF-IRF pathway without causing adverse effects associated with NF-κB inhibition, thereby offering advantages as a potential therapeutic agent for chronic inflammatory diseases.

## 3. Materials and Methods

### 3.1. General Procedures

Irradiation was carried out using an electron beam accelerator (10 MeV, Max. beam current of 3 mA, Max power of 30 kW, Model: MB10-30/3000) at the Advanced Radiation Institute (ARTI) of the Korea Atomic Energy Research Institute (Jeongup-si, Republic of Korea). NMR spectra were acquired with a VNS-600 Varian NMR spectrometer (^1^H NMR at 400 MHz, ^13^C NMR at 125 MHz, Palo Alto, CA, USA). HRCIMS was performed using a JMS-700 MStation Mass Spectrometer (JEOL Ltd., Tokyo, Japan). Optical rotation measurements were obtained using a JASCO DIP-1000 polarimeter (Tokyo, Japan), and ECD spectra were recorded with a JASCO J-1500 spectropolarimeter (Tokyo, Japan). The analytical HPLC-PDA analysis was conducted using a Nexera HPLC-PDA system (SHIMAZU Co., Kyoto, Japan) using an Agilent Eclipse XDB-C18 column (4.6 mm × 250 mm; 5 μM, Agilent Technologies, Inc., Santa Clara, CA, USA). Preparative HPLC was executed with a Waters system (Waters Corporation, Milford, CT, USA), incorporating a binary solvent delivery system and a PDA detector. Separation was performed using a YMC-Pack ODS-AQ column (10 × 250 mm; 5 μm, YMC Co., Tokyo, Japan).

### 3.2. Sample Preparation

Compound **1** (97.6% purity at 254 nm) was isolated by centrifugal partition chromatography from the supercritical carbon dioxide (SC-CO_2_) extract of leaves of *Perilla frutescens* var. *crispa* [30]. An amount of 1 mg of compound **1** was dissolved in 1 mL of water containing 10% DMSO and subjected to electron beam irradiation at absorbed doses of 40, 80, 120, 160, and 200 kGy for an individual scan. For bulk isolation of the radiolysis product, 110 mg of compound **1** was dissolved in 110 mL of water with 10% DMSO, divided into 55 vials and irradiated at 80 kGy. After electron beam irradiation, the samples were promptly evaporated to remove the solvent and then freeze-dried.

### 3.3. HPLC PDA Analysis

The electron beam-irradiated samples were analyzed using HPLC-PDA, equipped with an Eclipse XDB-C18 column (5 μm, 250 × 4.6 mm, Agilent Co., USA). The separation employed an isocratic solvent system of acetonitrile and water in a 40:60 ratio. The sample injection volume was set at 10 μL with a flow rate of 1 mL/min, while chromatograms were obtained at 254 nm utilizing a PDA detector.

### 3.4. Isolation of Isoegomaketone Derivative

The radiolysis product, compound **1** (110 mg; 99.0% purity at 254 nm) irradiated with 80 kGy electron beam, was purified using preparative HPLC with a YMC-Triart C18 ExRs column (5 μm, 250 × 20 mm, YMC Co., Tokyo, Japan). The separation used an isocratic mobile phase mixture of acetonitrile and water in 40:60 ratio. The flow rate was consistently maintained at 3 mL/min, and chromatograms were monitored at a wavelength of 254 nm. This process led to the isolation of radiolysis product **2**, which had a purity of 98.2% at 254 nm, yielding 4.19 mg which accounts for 3.80% of the output.

(±)-8-Methoxy-perilla ketone (**2**). Yellowish solid. ${\left[\alpha \right]}_{D}^{20}$ − 1.25 (*c* 0.08, CHCl_3_). ^1^H NMR (400 MHz, CDCl_3_) see Table 1. ^13^C NMR (125 MHz, CDCl_3_) δ 191.1 (C-6), 147.8 (C-5), 144.7 (C-2), 127.0 (C-3), 108.6 (C-4), 62.4 (C-8), 41.7 (OCH3), 34.7 (C-7), 26.7 (C-9), 21.4 (C-11), 17.8 (C-10). HRCIMS *m*/*z* 195.1023 [M–H]^−^, (calcd for C_11_H_15_O_3_, 195.1020).

### 3.5. Cytotoxicity Assay

Cell viability was evaluate using the EZ-Cytox assay kit. RAW 264.7 cells were cultured in a 96-well plate at a concentration of 2 × 10^5^ cells/mL for 24 h. Compound **2** was dissolved in DMSO and introduced to the cells at final concentrations of 5, 10, 20, and 40 µM, followed by an additional 24 h incubation. Subsequently, 10 μL of the cell viability reagent was added to each well, and the plates were incubated for 2 h at 37 °C in a 5% CO_2_ environment. The viability of the cell was assessed by measuring the formazan formation using a spectrophotometer at an absorbance of 480 nm, with 650 nm set as the reference wavelength.

### 3.6. Measurement of LPS-Induced NO Production on RAW 264.7 Cells

To measure NO production, RAW 264.7 cells were grown in culture. These cells were maintained in Dulbecco’s Modified Eagle’s Medium (DMEM), enriched with 10% fetal bovine serum (FBS), penicillin (100 units/mL), and streptomycin (100 μg/mL) at 37 °C in a humidified atmosphere with 5% CO_2_. The cells were seeded in 96-well plates and allowed to incubate for 24 h. Thereafter, they were pretreated with varying concentrations of the compound (5–40 μM) for 2 h, then stimulated with 1 μg/mL of LPS, in either the presence or absence of the test samples, for an additional 18 h. The culture medium was collected, and the nitrite level was determined as an indicator of NO production using the Griess reagent. This involved adding 100 μL of Griess reagent—containing 0.1% *N*-(1-naphthyl)ethylenediamine dihydrochloride in H_2_O and 1% sulfanilamide in 5% H_3_PO_4_—to 100 μL of each supernatant from the treated cells within 96-well plates. Absorbance reading was taken at 540 nm using a microplate reader, and nitrite concentrations were calculated using a sodium nitrite standard curve. The inhibition percentage of NO production was determined by the formula: [1 − (NO level of test samples/NO level of vehicle-treated control)] × 100. The IC_50_ value, representing the concentration required for 50% inhibition of NO production, was calculated by performing a non-linear regression analysis of % inhibition versus concentration.

### 3.7. Quantitative Real-Time PCR

RAW 264.7 cells were initially seeded in 96-well plates at a density of 2 × 10^5^ cells/mL and incubated for 24 h. Following this, the cells were treated with different concentrations of compound **2** for 2 h, and then stimulated with 1 μg/mL of LPS. For the analysis of iNOS mRNA, the cells underwent an additional 18 h of incubation after LPS stimulation, whereas for other cytokine mRNAs, incubation proceeded for 4 h. The cells were then harvested, and total RNA was extracted using RNeasy Kit (QIAGEN, Valencia, CA, USA). Complementary DNA (cDNA) was synthesized from the extracted RNA using a reverse transcription kit (Takara, Japan). Quantitative real-time PCR was conducted on the StepOnePlus Real-time PCR System (Applied Biosystems, Waltham, MA, USA). Each reaction was independently executed at least three times to ensure the reproducibility of results. Primers for iNOS, IFN-β, MCP-1, IL-1β, IL-6, and β-actin were acquired from Bioneer Corp. (Korea) (Daejeon, Republic of Korea), and PCR was performed on cDNA using the specified following forward and reverse primers: iNOS, forward primer, 5′-TCCTACACCACACCAAACTGTGTGC-3′, and reverse primer, 5′-CTCCAATCTCTGCCTATCCGTCTC-3′; IFN-β, forward primer, 5′-GGAAAGATTGACGTGGGAGA-3′, and reverse primer, 5′-GTGAGGCATCAACTGAACGG-3′; IL-1β, forward primer, 5′-GAGAATGACGTGTTCTTTGAAGTTGAC-3′, and reverse primer, 5′-TGAAGCTGGATGCTCTCATGAG-3′; IL-6, forward primer, 5′-ACAAAGCCAGAGTCCTTCAGAGAG-3′, and reverse primer, 5′-TTGGATGGTCTTGGTCCTTAGCC-3′; β-actin, forward primer, 5′-TGAGAGGGAAATCGTGCGTGAC-3′, and reverse primer, 5′-GCTCGTTGCCAATAGTGATGACC-3′.

### 3.8. Measurement of TNF-α, IL-6, and MCP-1 by ELISA

The concentrations of TNF-α, IL-6, and MCP-1 in the culture medium were determined using an ELISA kit (R&D systems, Hayward, CA, USA), following the manufacturer’s guidelines. Results are shown as the mean ± standard deviation (SD) of three replicates from one representative experiment.

### 3.9. Statistical Analysis

All experiments were carried out a minimum of three times in order to calculate means and standard deviations. Statistical significance was assessed using one-way analysis of variance (ANOVA) at a significance level of 0.05 after Shapiro-Wilk and Brown-Forsythe test for normality and equal variance. When ANOVA indicated a significant difference among groups, Tukey’s *post hoc* test was applied to compare the LPS-only treated group with those treated with compound **2**. Data analysis was conducted using GraphPad Prism9 (GraphPad Software, San Diego, CA, USA).

## 4. Conclusions

This study demonstrated that the radiolysis of isoegomaketone (**1**) in water containing 10% DMSO produced a structurally modified compound, (±)-8-methoxy-perilla ketone (**2**). Although compound **2** has previously been isolated from *P. fructescens* var. acuta and synthesized from the parent compound **1**, its reported NMR data were obtained using CCl_4_ as the NMR solvent, making direct comparison difficult. In the present study, detailed 2D NMR analysis using CDCl_3_ enables a clear assignment of the proton and carbon signals of **2**, providing a reliable reference for future elucidation. Compared to the parent compound **1**, compound **2** exhibited reduced inhibitory activity against LPS-induced NO production, but showed no cytotoxicity at high concentration. Further mechanistic studies revealed that compound **2** suppresses NO production via the TRIF-dependent IRF3 signaling pathway, whereas compound **1** acts through the MyD88-dependent NF-κB pathway. Taken together, these findings suggest that compound **2** may serve as a promising lead compound with both efficacy and safety for the treatment of inflammatory diseases. However, comprehensive in vivo validation studies, as well as pharmacokinetic and toxicological studies, will be necessary to further confirm the efficacy and safety of compound **2**.

## Figures and Tables

**Table 1:** Comparison of the ^1^H NMR data of compound **2** (CDCl_3_, 600 MHz) and 1-(3-furyl)-3-methoxy-4-methylpentan-1-one (CCl_4_) reported in Refs. [23,24].

Position	δ_H_, mult (*J* in Hz)	δ_H_, mult ^1^	δ_H_, mult ^2^
2	8.18, dd (1.2, 0.8)	8.00, s	8.14, s
4	6.83, dd (1.8, 1.2)	6.65, s	6.78, s
5	7.51, dd (1.8, 0.8)	7.40, s	7.49, s
7a	3.48, dd (18.6, 7.1)	2.72, d	2.5–3.1, m
7b	3.03, dd (18.6, 3.4)	2.72, d	2.5–3.1, m
8	3.84, dt (7.1, 3.4)	3.50, m	3.66, m
9	2.72, qqd (7.1, 3.4)	1.45, m	-
10 (Me)	1.1, d (7.1)	0.95, s	0.94, s
11 (Me)	1.06, d (7.1)	0.85, s	0.94, s
12 (OMe)	2.87, s	3.19, s	3.24, s

^1^ The ^1^H NMR data of 1-(3-furyl)-3-methoxy-4-methylpentan-1-one acquired in CCl_4_ [23]. ^2^ The ^1^H NMR data of 1-(3-furyl)-3-methoxy-4-methylpentan-1-one acquired in CCl_4_ [24], but the δ_H_ of H-9 was missing in this reference.

## Data Availability

The data presented in this study are available upon request from the corresponding authors.

## Supplementary Materials

The following supporting information can be downloaded at https://www.mdpi.com/article/10.3390/molecules30173466/s1. Figure S1: HPLC chromatograms of isoegomaketone (**1**) dissolved in water containing 10% DMSO, irradiated at five different doses (254 nm). (**A**) 0 kGy irradiation. (**B**) 40 kGy irradiation. (**C**) 80 kGy irradiation. (**D**) 120 kGy irradiation. (**E**) 160 kGy irradiation. (F) 200 kGy irradiation (for chromatography conditions, see Section 3.3); Figure S2: HPLC profiles (254 nm) of isoegomaketone (**1**) dissolved in methanol containing 10% DMSO irradiated with the following doses: 40, 80, 120, 160, and 200 kGy. (**A**) HPLC chromatogram aligned to Peak 1 (isoegomaketone, **1**). (**B**) HPLC chromatogram aligned to Peak 2 (radiolysis products of **1**) (for chromatography conditions, see Section 3.3); Figure S3: HPLC chromatograms of (**A**) compound **1** (a purity of 99.02%) and (**B**) compound **2** (a purity of 98.17%) at 254 nm (for chromatography conditions, see Section 3.3); Figure S4: HRCIMS spectrum of compound **2**; Figure S5: ^1^H NMR spectrum of compound **2**; Figure S6: ^13^C NMR spectrum of compound **2**; Figure S7: ^1^H-^1^H COSY spectrum of compound **2**; Figure S8: ^1^H-^13^C HSQC spectrum of compound **2**; Figure S9: ^1^H-^13^C HMBC spectrum of compound **2**; Figure S10: ECD spectrum of compound **2**; Figure S11: Cell viability and inhibitory effects on NO production of compounds **1** and **2**.
