# Supplementary material for: Ionizing Radiation-Induced Structural Modification of Isoegomaketone and Its Anti-Inflammatory Activity
Source: Molecules. 2025 Aug 23;30(17):3466. doi: 10.3390/molecules30173466 (PMC12430447; doi:10.3390/molecules30173466)
Supplement: Supplementary file 1 [file molecules-30-03466-s001.zip › molecules-3793585-supplementary.pdf]

Supporting Information

Ionizing Radiation-Induced Structural Modification of Isoegomaketone and Its Anti-Inflammatory Activity

Euna Choi [1,†], Chang Hyun Jin [1,†], Trung Huy Ngo [2], Jisu Park [1], Joo-Won Nam [2,*] and Ah-Reum Han [1,*]

1 Advanced Radiation Technology Institute, Korea Atomic Energy Research Institute, Jeongeup-si, Jeollabuk-do 56212, Republic of Korea
2 College of Pharmacy, Yeungnam University, Gyeongsan-si, Gyeongsangbuk-do 38541, Republic of Korea
* Correspondence: arhan@kaeri.re.kr (A.-R.H.); jwnam@yu.ac.kr (J.-W.N.)
† These authors contributed equally to this work

CONTENTS

Figure S1. HPLC chromatograms of isoegomaketone (**1**) dissolved in water containing 10% DMSO, irradiated at five different doses (254 nm). (**A**) 0 kGy irradiation. (**B**) 40 kGy irradiation. (**C**) 80 kGy irradiation. (**D**) 120 kGy irradiation. (**E**) 160 kGy irradiation. (F) 200 kGy irradiation (for chromatography conditions, see Section 3.3).

Figure S2. HPLC profiles (254 nm) of isoegomaketone (**1**) dissolved in methanol containing 10% DMSO irradiated with the following doses: 40, 80, 120, 160, and 200 kGy. (**A**) HPLC chromatogram aligned to Peak 1 (isoegomaketone, **1**). (**B**) HPLC chromatogram aligned to Peak 2 (radiolysis products of **1**) (for chromatography conditions, see Section 3.3).

Figure S3. HPLC chromatograms of (**A**) compound **1** (a purity of 99.02%) and (**B**) compound **2** (a purity of 98.17%) at 254 nm (for chromatography conditions, see Section 3.3).

Figure S4. HRCIMS spectrum of compound **2**

Figure S5. ^{1}H NMR spectrum of compound **2**

Figure S6. ^{13}C NMR spectrum of compound **2**

Figure S7. ^{1}H-^{1}H COSY spectrum of compound **2**

Figure S8. ^{1}H-^{13}C HSQC spectrum of compound **2**

Figure S9. ^{1}H-^{13}C HMBC spectrum of compound **2**

Figure S10. ECD spectrum of compound **2**

Figure S11. Cell viability and inhibitory effects on NO production of compounds **1** and **2**.

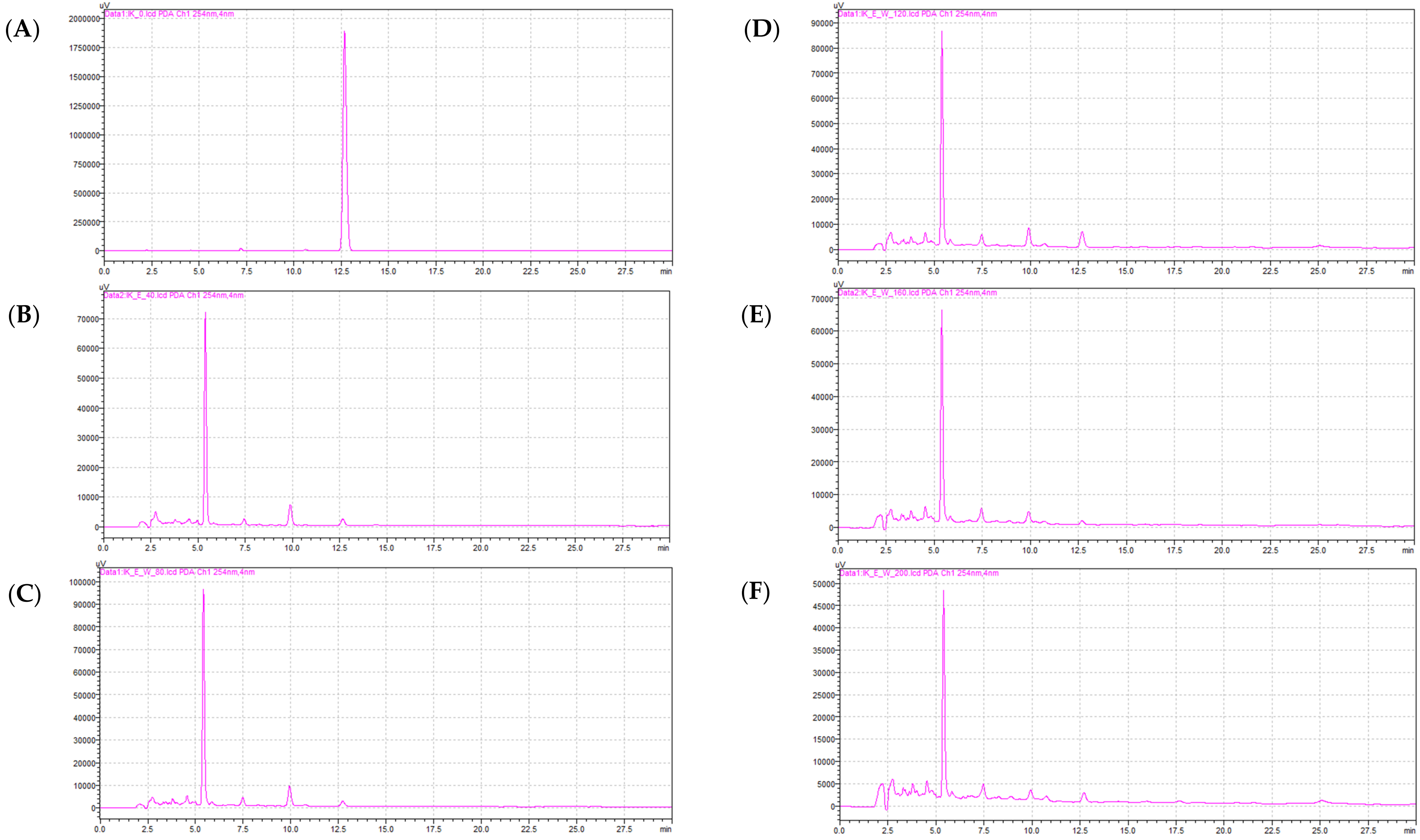

Figure S1. HPLC chromatograms of isoegomaketone (**1**) dissolved in water containing 10% DMSO, irradiated at five different doses (254 nm). (**A**) 0 kGy irradiation. (**B**) 40 kGy irradiation. (**C**) 80 kGy irradiation. (**D**) 120 kGy irradiation. (**E**) 160 kGy irradiation. (F) 200 kGy irradiation (for chromatography conditions, see Section 3.3).

(A)
(B)

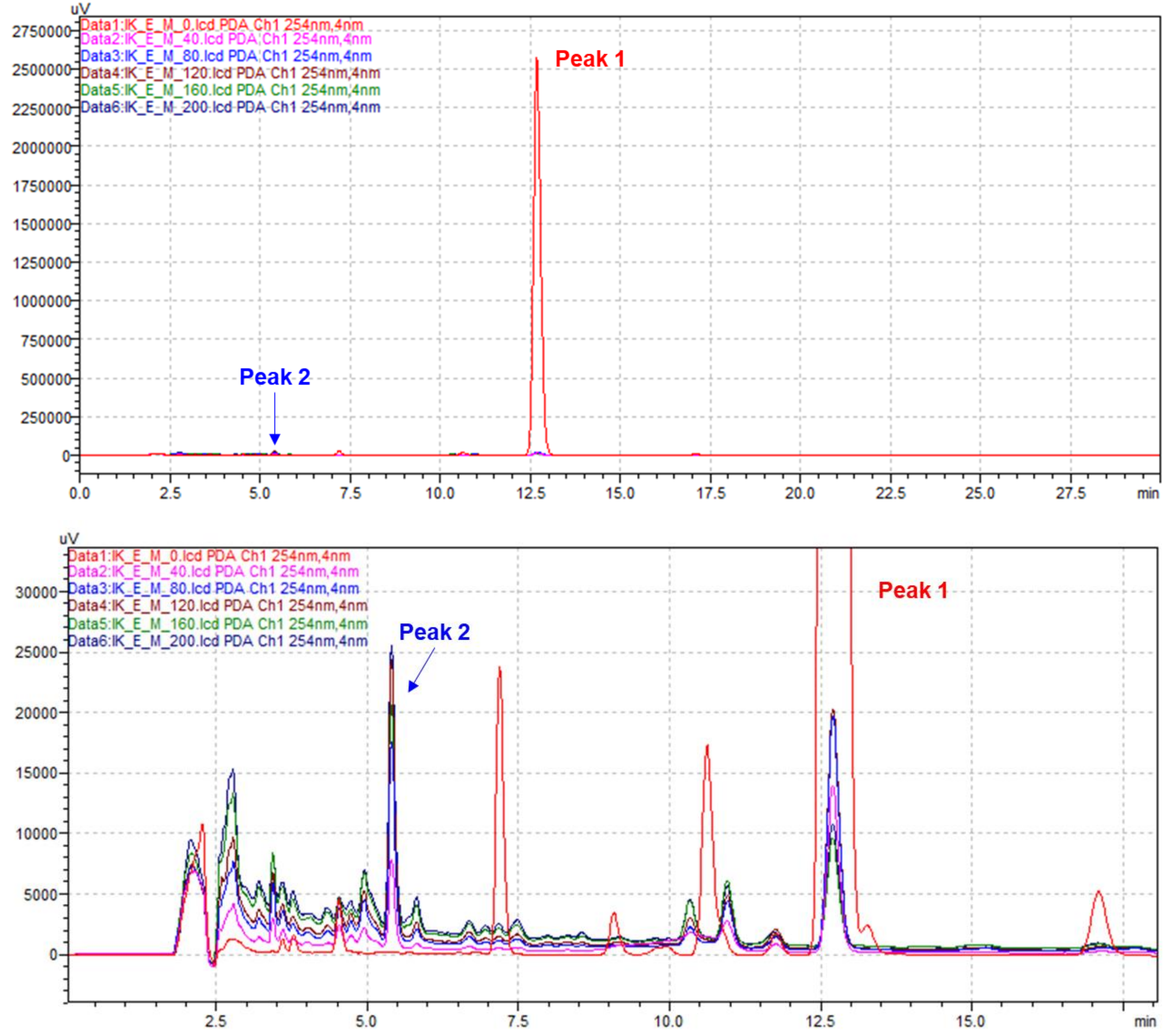

Figure S2. HPLC profiles (254 nm) of isoegomaketone (**1**) dissolved in methanol containing 10% DMSO irradiated with the following doses: 40, 80, 120, 160, and 200 kGy. (**A**) HPLC chromatogram aligned to Peak 1 (isoegomaketone, **1**). (**B**) HPLC chromatogram aligned to Peak 2 (radiolysis products of **1**) (for chromatography conditions, see Section 3.3).

(**A**)

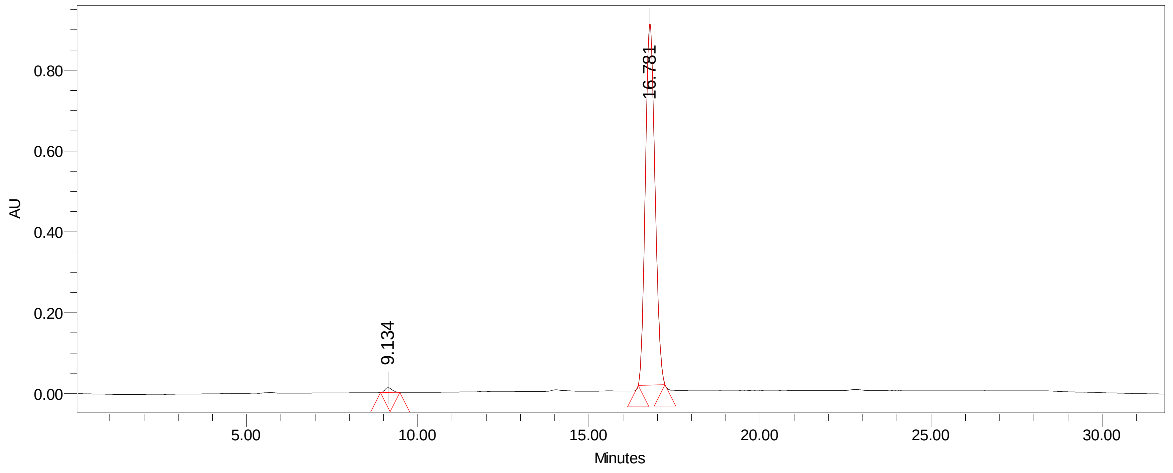

	Name	Retention Time (min)	Purity1 Angle	Purity1 Threshold	PDA/FLR Match1 Spect. Name	PDA/FLR Match1 Angle	PDA/FLR Match1 Threshold	PDA/FLR Match1 Lib. Name	Area (µV*sec)	% Area	Height (µV)	Int Type
1		9.134							172722	0.98	11968	bb
2		16.781							17495868	99.02	895139	bb

(**B**)

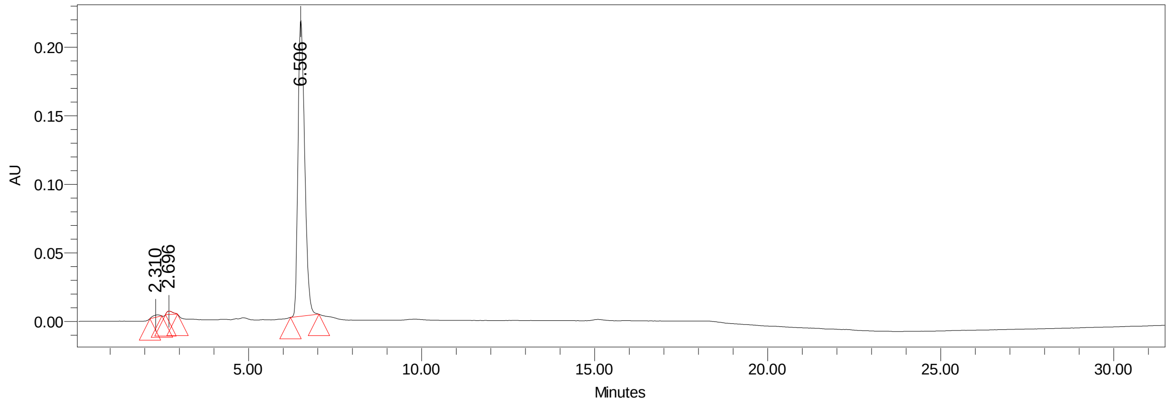

	Name	Retention Time (min)	Purity1 Angle	Purity1 Threshold	PDA/FLR Match1 Spect. Name	PDA/FLR Match1 Angle	PDA/FLR Match1 Threshold	PDA/FLR Match1 Lib. Name	Area (µV*sec)	% Area	Height (µV)	Int Type
1		2.310							22894	0.77	1655	bb
2		2.696							31278	1.05	2521	bb
3		6.506							2913011	98.17	215922	bb

Figure S3. HPLC chromatograms of (**A**) compound **1** (a purity of 99.02%) and (**B**) compound **2** (a purity of 98.17%) at 254 nm (for chromatography conditions, see Section 3.3).

[Mass Spectrum]
Data : CI959 Date : 24-Mar-2025 14:47
RT : 1.55 min Scan# : (49,84)
Elements : C 100/0, H 100/0, O 10/0
Mass Tolerance : 10ppm, 5mmu if m/z < 500, 10mmu if m/z > 1000
Unsaturation (U.S.) : -0.5 - 20.0

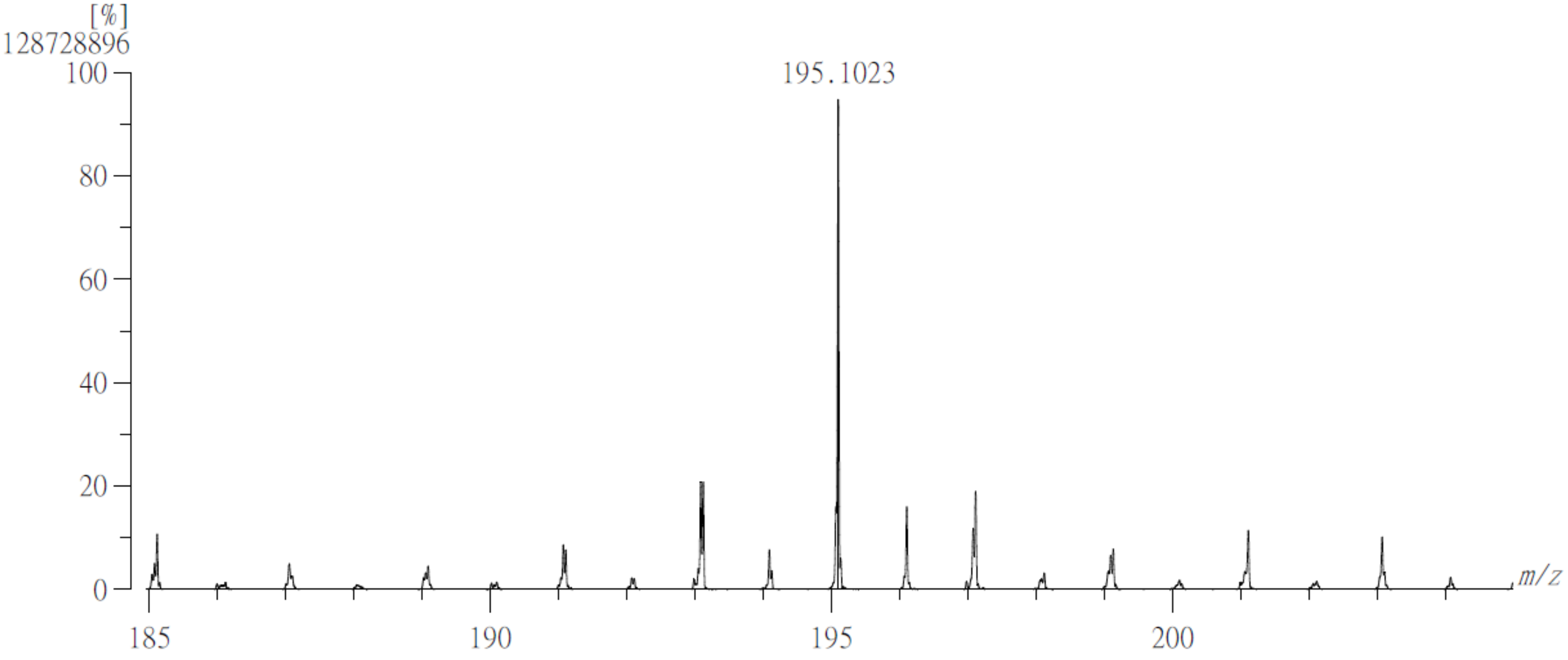

	Observed m/z	Int%	Err[ppm / mmu]	U.S.	Composition
1	195.1023	94.71	+0.9 / +0.2	4.5	C11 H15 O3

Figure S4. HRCIMS spectrum of compound **2**.

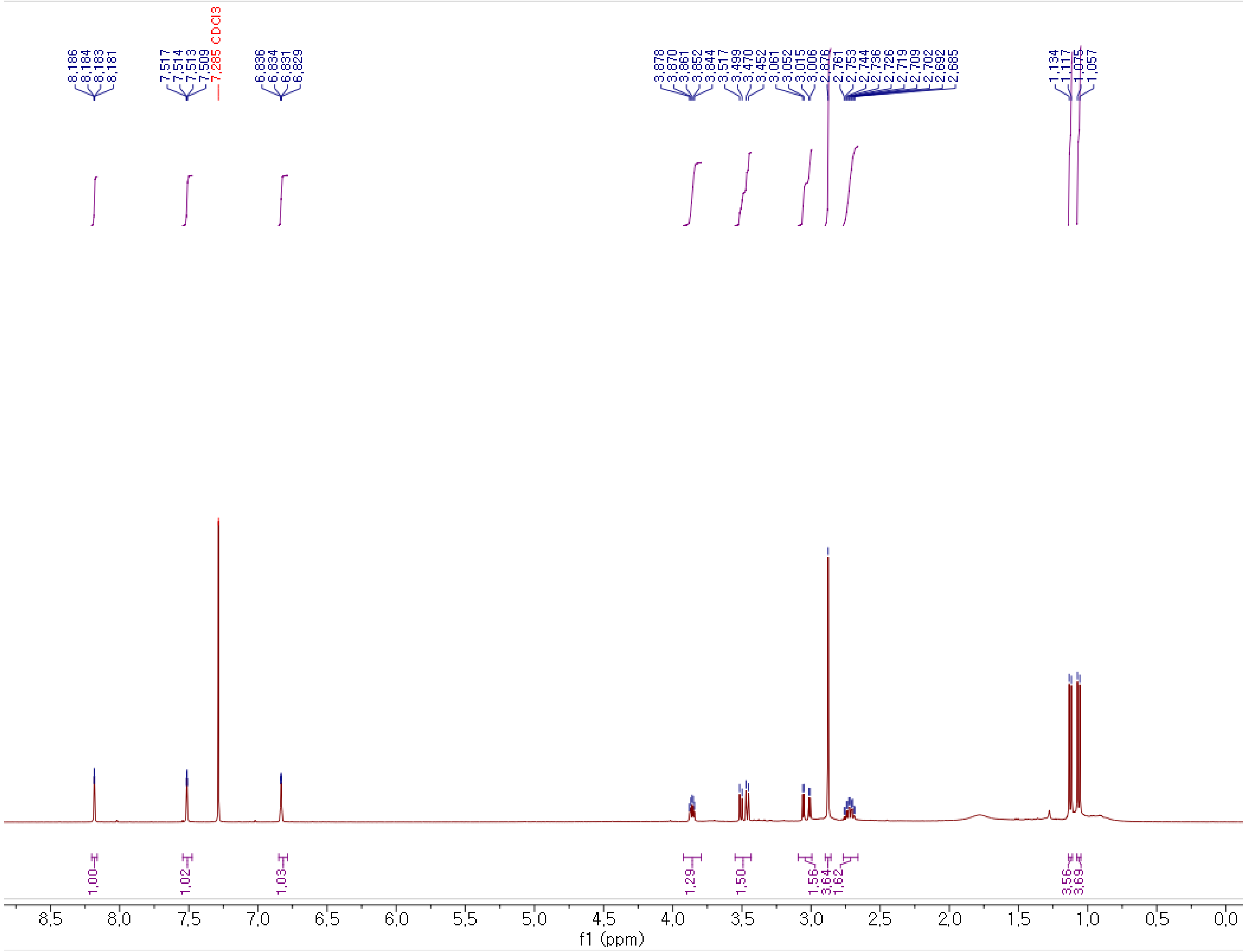

Figure S5. ^{1}H NMR spectrum of compound **2**.

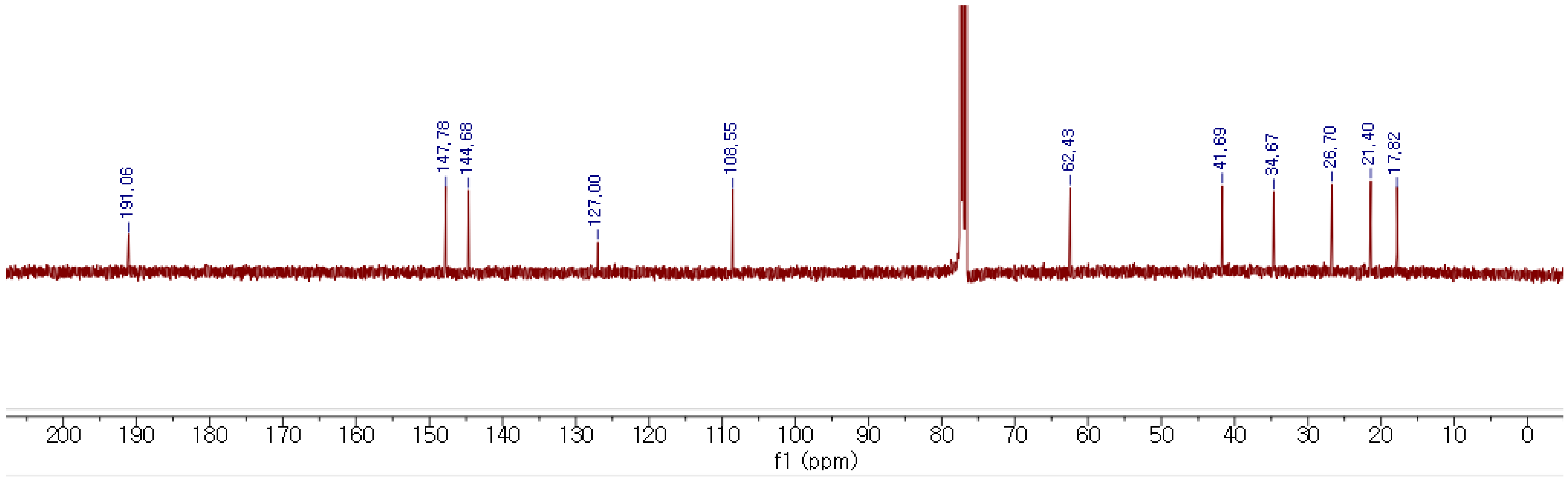

Figure S6. ^{13}C NMR spectrum of compound **2**.

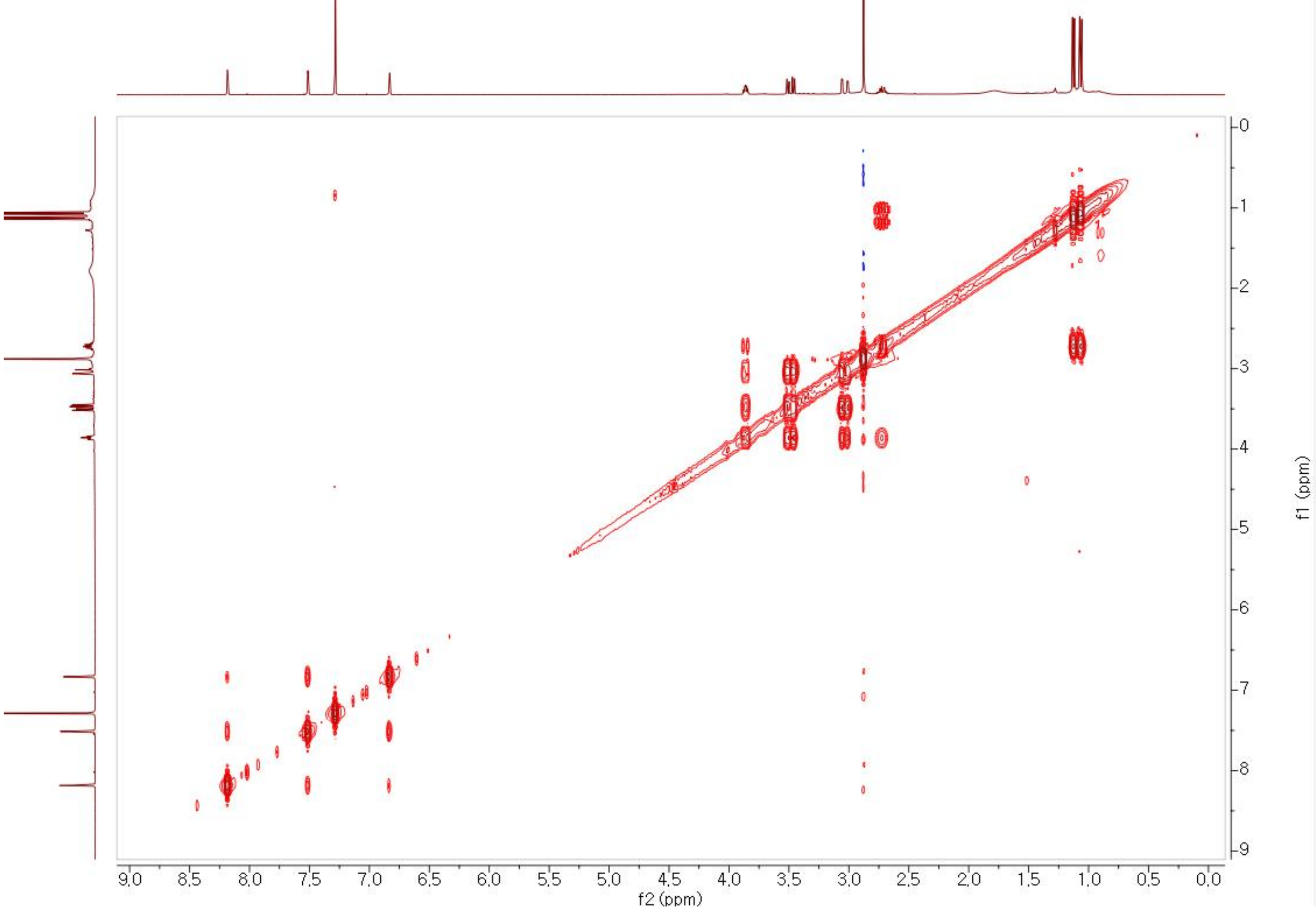

Figure S7. ^{1}H-^{1}H COSY spectrum of compound **2**.

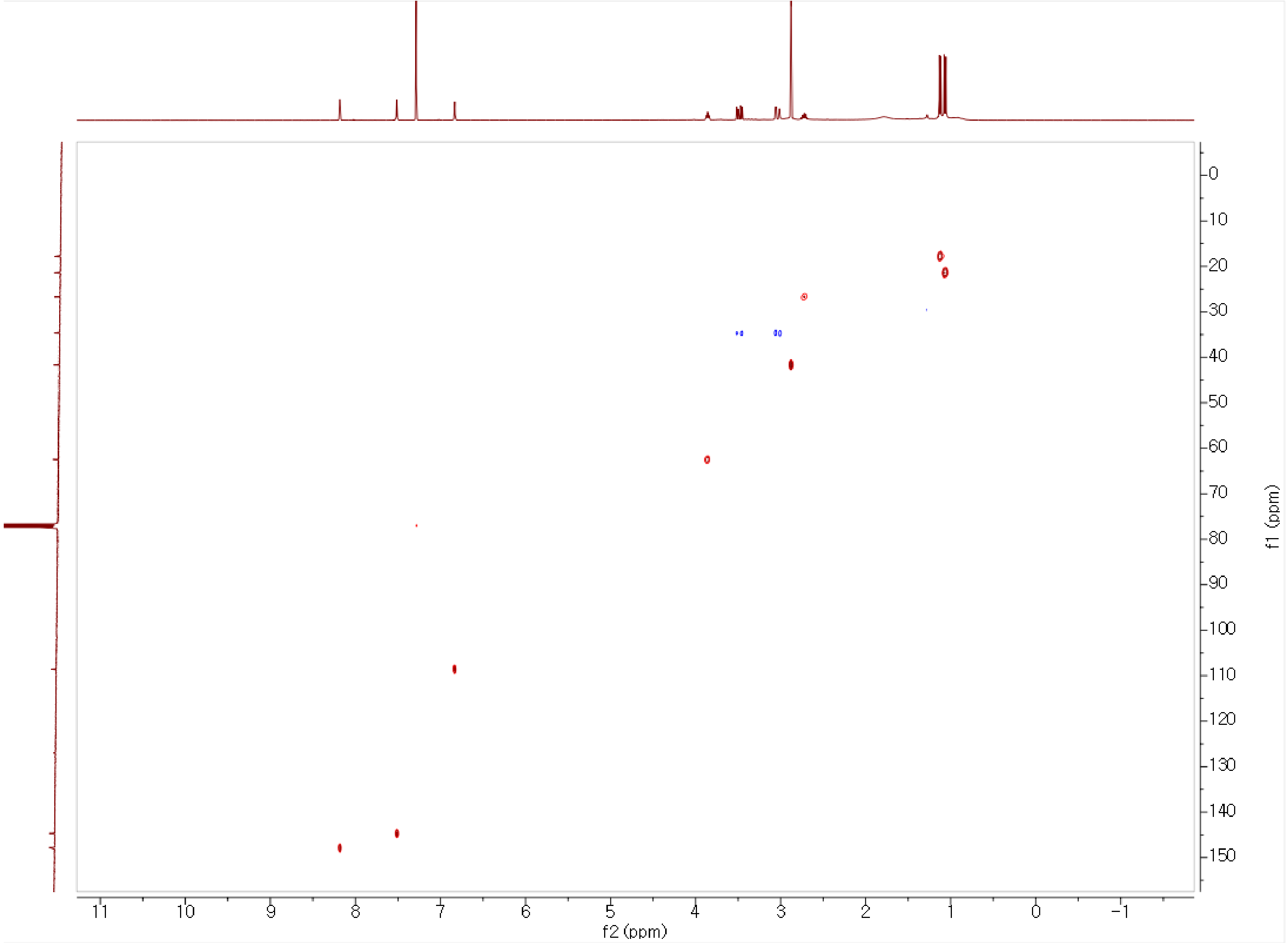

Figure S8. ^{1}H-^{13}C HSQC spectrum of compound **2**.

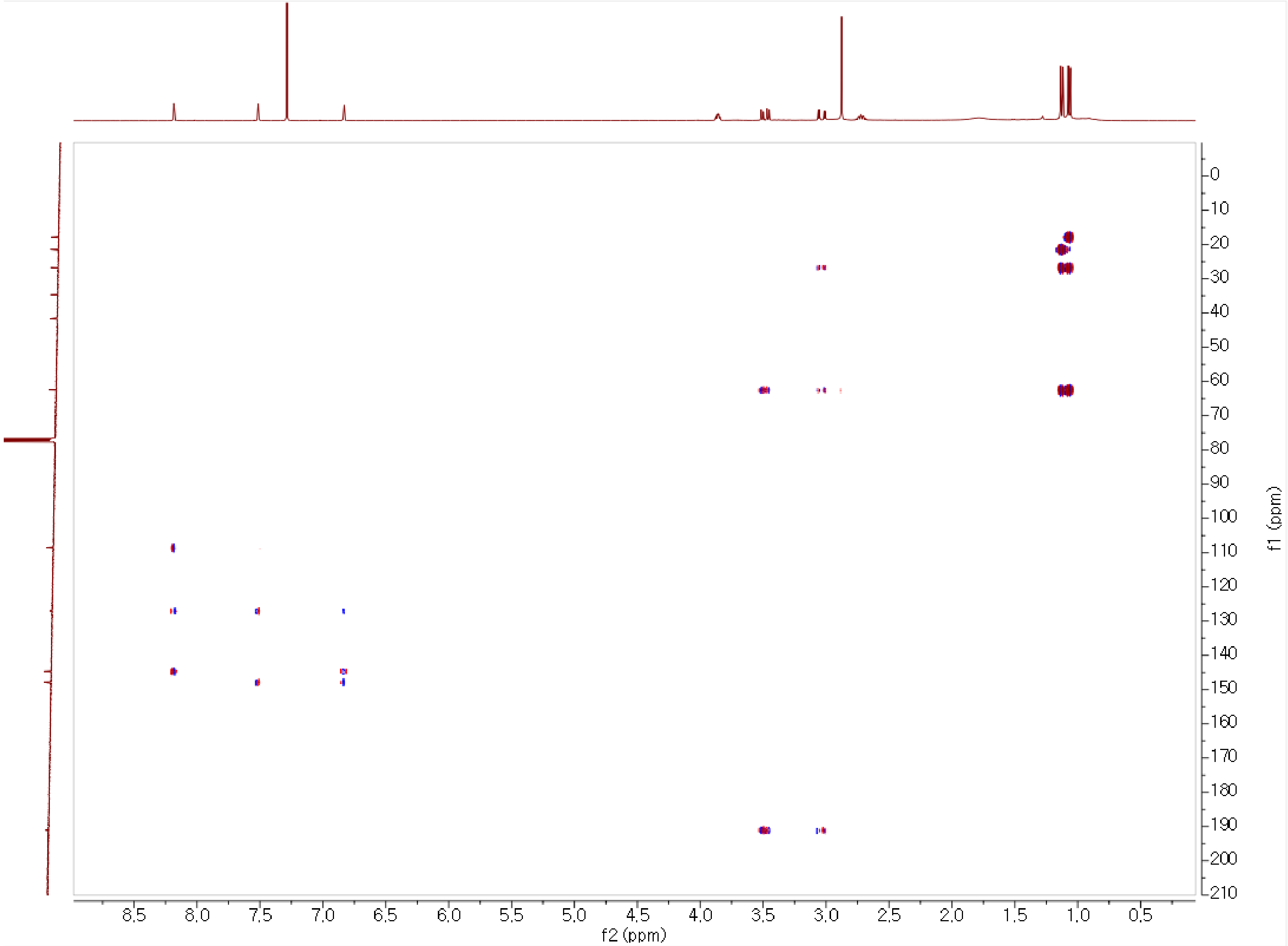

Figure S9. ^{1}H-^{13}C HMBC spectrum of compound **2**.

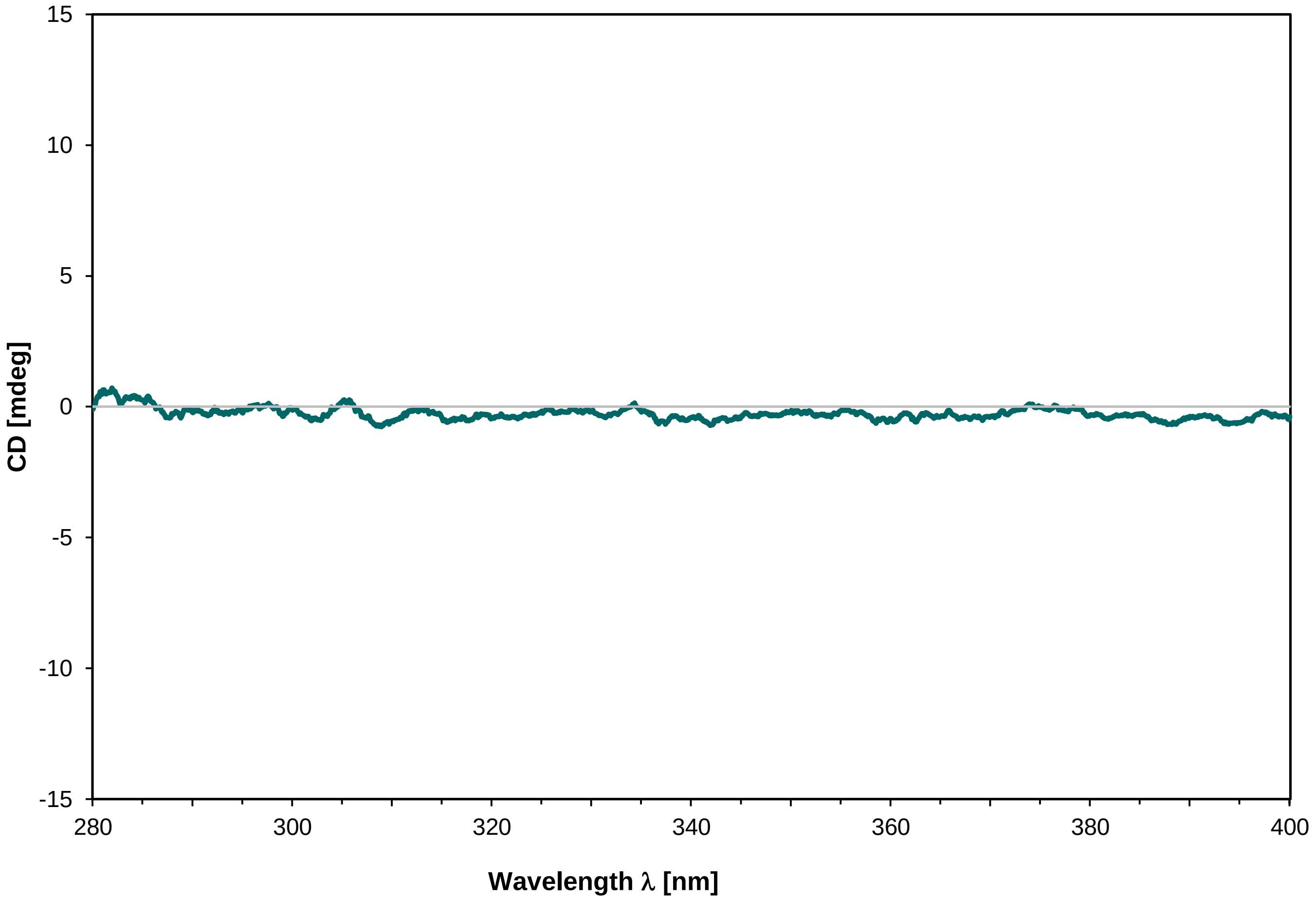

Figure S10. ECD spectrum of compound **2**.

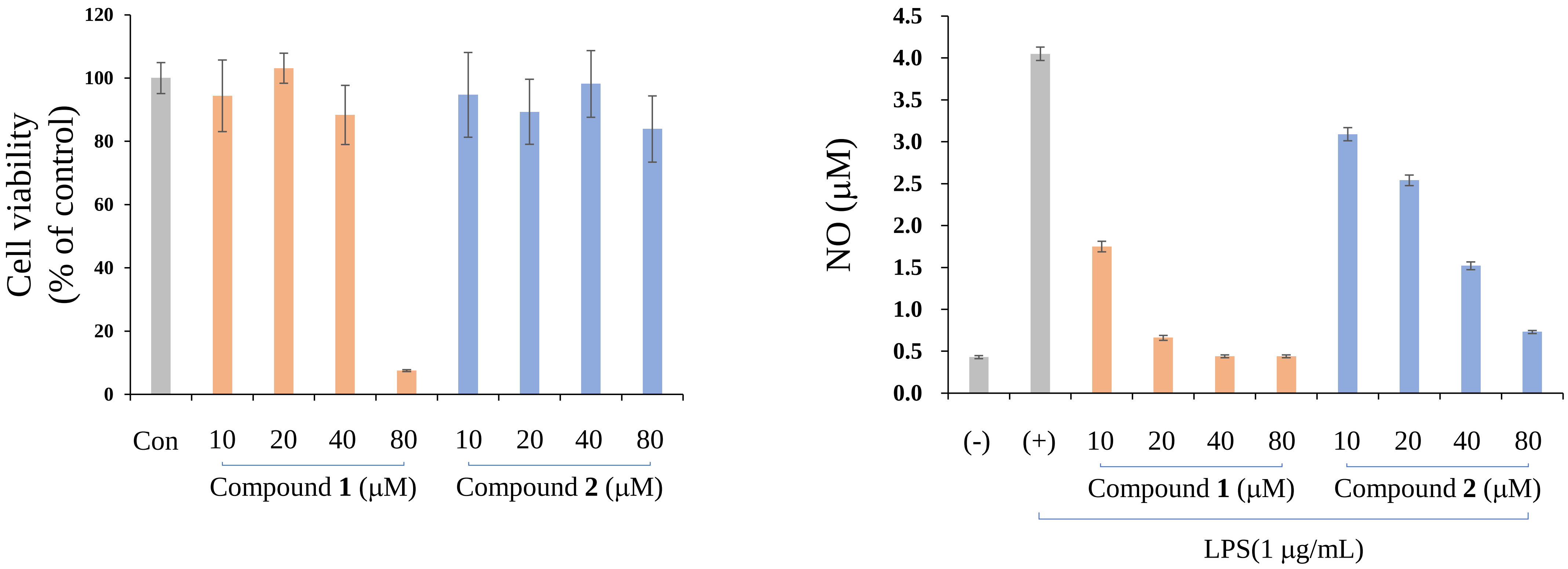

Figure S11. Cell viability and inhibitory effects on NO production of compounds **1** and **2**.
